# Latent goal models for dynamic strategic interaction

**DOI:** 10.1371/journal.pcbi.1006895

**Published:** 2019-03-11

**Authors:** Shariq N. Iqbal, Lun Yin, Caroline B. Drucker, Qian Kuang, Jean-François Gariépy, Michael L. Platt, John M. Pearson

**Affiliations:** 1 Duke Institute for Brain Sciences, Center for Cognitive Neuroscience, Duke University, Durham, NC, USA; 2 Departments of Neuroscience, Marketing, and Psychology, University of Pennsylvania, Philadelphia, PA, USA; 3 Department of Biostatistics and Bioinformatics, Duke University, Durham, NC, USA; Brain and Spine Institute (ICM), FRANCE

## Abstract

Understanding the principles by which agents interact with both complex environments and each other is a key goal of decision neuroscience. However, most previous studies have used experimental paradigms in which choices are discrete (and few), play is static, and optimal solutions are known. Yet in natural environments, interactions between agents typically involve continuous action spaces, ongoing dynamics, and no known optimal solution. Here, we seek to bridge this divide by using a “penalty shot” task in which pairs of monkeys competed against each other in a competitive, real-time video game. We modeled monkeys’ strategies as driven by stochastically evolving goals, onscreen positions that served as set points for a control model that produced observed joystick movements. We fit this goal-based dynamical system model using approximate Bayesian inference methods, using neural networks to parameterize players’ goals as a dynamic mixture of Gaussian components. Our model is conceptually simple, constructed of interpretable components, and capable of generating synthetic data that capture the complexity of real player dynamics. We further characterized players’ strategies using the number of change points on each trial. We found that this complexity varied more across sessions than within sessions, and that more complex strategies benefited offensive players but not defensive players. Together, our experimental paradigm and model offer a powerful combination of tools for the study of realistic social dynamics in the laboratory setting.

## Author summary

Most studies of strategic decision making make use of simple tasks in which agents choose among only a limited number of distinct options. But real-world behavior is complex, requiring ongoing adjustment of strategies. Here, we propose a new model that is capable of reproducing the rich behavior of monkeys playing against each other in a dynamic decision task. Our model quantifies players’ goals at each moment and offers a means of performing controlled experiments via simulation. This makes possible more realistic experimental paradigms for the study of strategic decision making and social interaction.

## Introduction

Humans are a social species. Our most difficult and most crucial decisions—whom to trust, whom to double-cross, with whom to rear children—are most often decisions about other agents. Indeed, it has been conjectured that group living formed the primary driving force in the evolution of human cognition [1]. Yet social decisions are also among the most complex that agents face, since the relevant costs and benefits rely not only on one’s own preferences and options, but on others’ preferences, their options, and (potentially) their assessments of one’s intentions [2, 3].

Over the last decade, much neuroscientific work has attempted to understand the physiological and cognitive basis for these social decisions [3–5]. Most of these studies have been performed in humans using non-invasive methods like functional MRI and EEG, though an important (and growing) strand of work has used animal models [6–14], which allow for direct neural recording. In both cases, the behavioral tool of choice has been game theory. Game theory offers several important benefits for the study of social decisions: models of both cooperative and competitive interaction; a rich, well-developed theory; and a clear notion of optimality, Nash equilibrium, against which subjects’ behavior can be compared. In addition, there are vast parallel literatures studying empirical game play in real agents [2], evolutions of strategies [15], and games as models for ecological behavior [16].

Nonetheless, such paradigms are also limited as models of real-world social decisions. For example, in typical paradigms, the choice space is discretized and low-dimensional, whereas real social decisions are embodied, requiring movement in space from multiple effectors. Moreover, because laboratory tasks often involve (repeated) single decisions among small numbers of alternatives, the space of available strategies is highly constrained, often isomorphic to the space of choices. By contrast, interaction in social settings involves feedback from other agents in real-time, with a commensurately large space of potential strategies. Unfortunately, moving beyond small numbers of discrete choices drastically complicates behavioral analysis. Optimality may no longer be well-defined, and when it is, finding a solution may yet be computationally intractable. Moreover, when the potential behavioral repertoire on each bout of play is large, it may be difficult, if not impossible, to meaningfully average physiological signals like neural activity across bouts.

Here we use insights from generative modeling, control theory, and inverse reinforcement learning [17, 18] to show how it is nonetheless possible to characterize strategies in a dynamic, competitive decision at the single-trial level. Using data collected from a laboratory task in which pairs of rhesus macaques used joysticks to control onscreen avatars, we describe the avatars’ resulting trajectories in terms of time-varying latent “goals”—onscreen locations that function as set points for a control model that produces joystick movement. We use methods of scalable, approximate Bayesian inference to both infer goal trajectories at the single-trial level and generate entirely new gameplay that captures the richness of real player behavior. In the model, goals themselves are generated by a stochastic process analogous to the motion of a particle in a potential energy well, with the potential energy term capturing the interaction of each player’s goals with his opponent’s observed actions. This potential energy, flexibly parameterized by neural networks, allows us to succinctly describe player interactions apart from details of the control process, providing moment-by-moment measures of intention and decision complexity that can be correlated with neural signals. Just as importantly, though the model is developed for the case of a simple screen-based task with two players, it easily generalizes to other time series data (including >2 real or artificial agents) in which observations are the result of a control process and latent goals are the underlying variables of interest.

In the sections below, we first describe the two-player dynamic decision task and its attendant data. We then outline our generative modeling assumptions, including the control and latent goal models. After that, we describe how to perform approximate Bayesian inference in the model using scalable Variational Bayes methods [19]. We assess the outputs of the model and compare with simpler alternatives, showing that the model not only captures the rich variation present in real game play but is capable of generating novel data of similar complexity. We then demonstrate that goals are better predictors of trial outcomes than either current velocity or players’ gaze positions and give an example of how our model can be used to perform controlled experiments via simulation of novel data. Finally, we use a simple measure of strategic complexity to consider the effect of this quantity on win rate. We conclude by discussing applications of our model to neural data analysis.

## Methods and models

### Subjects, task, and data

#### Ethics statement

All protocols and procedures were approved by the Duke University Institutional Animal Care and Use Committee.

#### Subjects

Three adult male rhesus macaques (monkeys O, Y, and E) played as shooters in the penalty shot game described below, and these three plus one other (monkey D) played as goalies. All subjects were singly housed, and were kept on a water-restricted diet to increase motivation to earn liquid reward in the experimental task.

#### Task

Two monkeys sat in primate chairs (Crist Instruments, Damascus, MD) modified to allow access to joysticks (IP66s, CTI Electronics, Stratford, CT) affixed in a custom-built acrylic case at the front. All axes of the joystick were calibrated with a MATLAB (Natick, MA, USA) script to ensure that every direction yielded equal speed. Horizontal and vertical eye positions of the shooter were sampled at 1000 Hz by an infrared camera (Eyelink 1000, SR Research, Kanata, Ontario, Canada). The task was presented and data were collected via custom-built scripts in MATLAB using the Psychophysics and Eyelink Toolbox extensions [20] (see http://psychtoolbox.org/). Primate chairs were oriented at a ninety-degree angle to one another such that each monkey could see the other to his left or right. Each monkey also faced his own computer monitor displaying the task; the display was the same on both screens.

**Fig 1:**
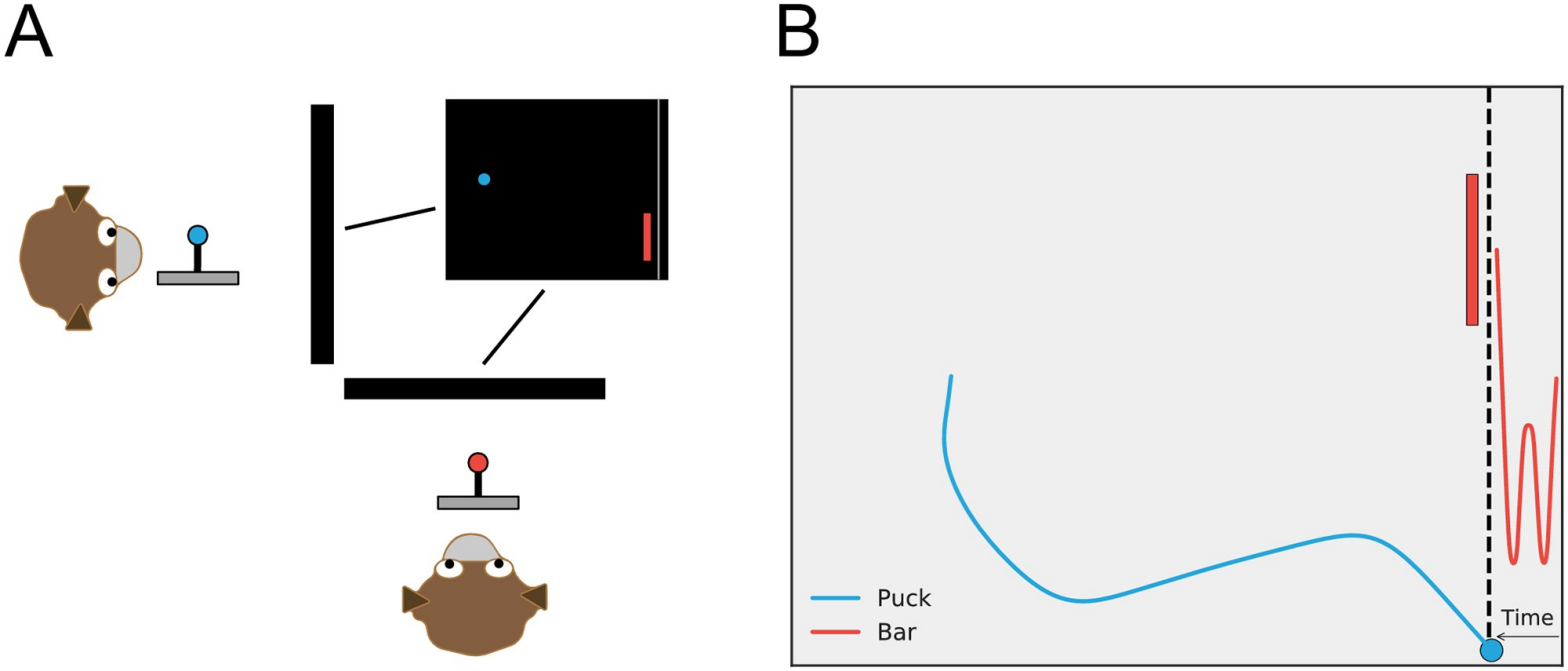
Penalty shot task and game play. A: The two subjects viewed the same image on separate screens. One subject (at left here) played as the “shooter” and controlled the puck, while the other (at bottom here) played as the “goalie” and controlled the bar. B: Illustration of onscreen play for the two-player game. The puck (blue) is free to move in two dimensions, while the goalie (red) moves only up or down. Axes are x and y screen directions. Solid lines indicate player trajectories for a single trial. The goalie’s trajectory has been stretched along the x axis to indicate its movement through time. For animations of game play, see S1, S2, S3 and S4 Videos.

Each trial was preceded by a 300ms period during which the monkeys each had to center their joysticks. Then a green circle, the puck, appeared at the left side of the screen, centered vertically. A long red rectangle, the goalie bar, appeared at the vertical center near the right of the screen. Further to the right, a gray line, the goal, extended from the top to the bottom of the screen. The screen was otherwise black (Fig 1). The “shooter” monkey’s joystick controlled the movement of the puck, which was unconstrained in all directions except that it could not go beyond the borders of the screen, and the “goalie” monkey’s joystick controlled the movement of the bar, which was similarly unconstrained vertically but did not move horizontally. The monkeys’ roles were fixed throughout an experimental session. Each trial ended when the puck reached the goal, when the puck hit the goalie bar, or when ten seconds had elapsed with no movement from the shooter. The horizontal and vertical location of the puck, as well as the vertical location of the goalie bar, were recorded throughout the trial at roughly 60 Hz. If the puck reached the goal, the shooter was considered to have won and he received a drop of juice in his mouth. If the puck hit the goalie bar, the goalie was considered to have won and he received a drop of juice in his mouth. Juice volume on each successful trial was similar for both monkeys and constant throughout the session. Following juice delivery, the screen went blank for a 1.5 s inter-trial interval before the next trial began. The speeds of the puck and goalie bar were titrated by the experimenter between gaming blocks (roughly 200 trials) to achieve a roughly 50% win rate for each player in each session. Monkeys were first trained against a computer opponent performing simple movements and subsequently confronted monkey opponents.

#### Data

Data consisted of puck and goalie trajectories on each trial for *N* = 120 behavioral sessions (Shooter sessions: Monkey O: 59, Monkey Y: 55, Monkey E: 6) for a total of 59,884 trials. For demonstrating model fits and generated trials, we also used a smaller, more homogeneous dataset comprising 8659 trials from the last 10 sessions of the experiment (all Monkey O).

Each trial’s data comprised a three-dimensional vector *y* = (*x*_puck_, *y*_puck_, *y*_goalie_) at each time, sampled at a rate of approximately 60 samples per second. Prior to analysis, data were preprocessed by independently normalizing the range of each coordinate in *y* to [−1, 1]. To accurately estimate conditions at the beginning of each trial, we smoothed data using a Gaussian kernel with length *w* = 41 and *σ* = 4. Nonetheless, this smoothing is affected by a bias-variance tradeoff. To minimize bias in the initial condition, the first $\left\lfloor \frac{w}{2}\right\rfloor$ time points were reflected in time and sign and prepended to the data:

$$\begin{array}{c} y_{-t}=2y_{1}-y_{1+t} \end{array}$$ (1)

so that *y*_1_ remained invariant under the smoothing. Similarly, to avoid introducing artifacts in control at the end of the trial, data were linearly extrapolated past the last time point for smoothing. Alternatives to this procedure yielded smoother trajectories at the cost of greater bias in initial conditions, including initial velocity.

For model fitting, data were augmented by two additional variables at each time point. The first variable encoded a linear trend that was fixed at the session level and ranged from 0 (first session of the experiment) to 1 (last session). The second variable was a binary indicator corresponding to the brain region in which electrophysiological recordings were performed for the session in which the trial took place.

Finally, we used gaze data collected from shooter monkeys. Data were sampled at 1kHz and reinterpolated at 900Hz, such that each behavioral time stamp corresponded to 15 gaze samples.

### Game dynamics

Let *y*_*t*_ be the observed variables of the onscreen trajectory at time *t*. Similarly, let *s*_*t*_ be the collection of system variables. We typically consider the latter to be positions and velocities (i.e., $s_{t}=\left(y_{t},{\dot{y}}_{t}\right)$), but they could additionally include accelerations, additional time points of lagged data, or variables encoding player identity or game condition. For the penalty shot task, *y*_*t*_ evolves according to

$$\begin{array}{c} y_{t+1}=y_{t}+v_{\text{max}}⊙\upsilon_{t} \end{array}$$ (2)

with *v*_max_ a vector of maximum velocities in each dimension as determined from the data, *υ* ∈ [−1, 1] a joystick position, and ⊙ the Hadamard (elementwise) product. We further assume that the joystick position *υ* is related to a *latent* control signal *u* via *υ* = tanh(*u*). That is, the observed control, restricted by the finite joystick range, may be less than desired control.

### Control model

We assume that at each time *t*, *u*_*t*_ is the output of a control model attempting to minimize an error *e*_*t*_ ≡ *g*_*t*_−*y*_*t*_ with *g*_*t*_ an instantaneous, state-dependent, set point for the controller that we will call a “goal”. These goals represent the instantaneous onscreen locations desired by each player, as evidenced by the fact that when *y*_*t*_ = *g*_*t*_(*s*_*t*_) (next goal and position are equal), we have *e*_*t*_ = 0 and thus no need for control. When *e*_*t*_ ≠ 0, however, we assume that changes in control are given by a proportional-integral-derivative (PID) controller:

$$\begin{array}{cc} \Delta u_{t} & \equiv u_{t}-u_{t-1}=L*e_{t}=\sum_{\tau =0}^{2}L_{\tau }e_{t-\tau }\\ & =\kappa [(1+\frac{\Delta t}{T_{i}}+\frac{T_{d}}{\Delta t})e_{t}+(-1-\frac{2T_{d}}{\Delta t})e_{t-1}+\frac{T_{d}}{\Delta t}e_{t-2}] \end{array}$$ (3)

with *κ* the proportional control constant and *T*_*i*_ and *T*_*d*_ the integration and differentiation time constants, respectively. That is, *L* is a convolutional filter, determined by *κ*, *T*_*i*_, and *T*_*d*_, learned separately for each player in each dimension. Changes in control are thus convolutions over the control error. Finally, to capture our uncertainty about this relationship, we model errors in control as normally distributed with variance *ϵ*^2^:

$$\begin{array}{c} u_{t}∼N\left(u_{t-1}+L*\left(g_{t}-y_{t}\right),\epsilon^{2}\right) \end{array}$$ (4)

where the equation should be read as implying a separate, uncorrelated, normal distribution for each coordinate in *u* (with potentially distinct *ϵ*_*i*_ in each dimension). In practice, our onscreen observations have minimal noise, implying *ϵ* ≪ 1.

Unfortunately, Eq 4 is ambiguous, since for any sequence of controls *u*_*t*_ and any *L*, the equations for *g* are linear and thus in general invertible. And while this symmetry is weakly broken by our model for *g* (described below), which prefers some sequences of goals to others, we remove the ambiguity in practice by fixing control to be purely proportional early in training, consistent with the idea of a goal as a point toward which each player desires to move.

In addition, a second issue arises from the fact that joystick positions are constrained to lie in [−1, 1] along each dimension, while model-predicted control might be large. In our model, we achieve this by using a hyperbolic tangent function to link *u* (desired control) to *υ* (joystick input). However, this means that small changes in *υ* can result in potentially large changes in inferred *u* and thus *g*. More concretely, when joystick input is near maximal (*υ* ≈ ±1), this will be consistent for any goal *farther* than a certain distance from the player’s current location. Ideally, one would consider *υ* a censored version of *u*, a possibility we leave to future work. Here, we remedy this by imposing a penalty during model training on any goals that far exceed the visible game area.

### Goal model

For the goal time series, *g*_*t*_, we will assume a Markov process in which new goals are probabilistically selected at each time based on both the current goal and the current state of the system. That is,

$$\begin{array}{c} p\left(u,g\right)\propto \prod_{t}p\left(u_{t}|u_{t-1},g_{t},y_{t}\right)p\left(g_{t}|g_{t-1},s_{t}\right) \end{array}$$ (5)

More specifically, we will assume that at each time point, there exists a function *V*(*g*_*t*_, *s*_*t*_) that captures the benefit in setting a particular goal based on the current state of the system. That is, we want to increase *V* as often as possible. (Alternately, we could consider −*V* as an energy function that we wish to minimize.) However, we also assume that the goal time series is relatively smooth, which we formalize by adding a regularization term for the rate of change between successive time points. More explicitly, let

$$\begin{array}{c} \log p\left(u,g\right)=\sum_{t}[-\frac{1}{2\epsilon^{2}}{∥u_{t}-u_{*t}\left(g_{t},y_{t}\right)∥}^{2}-\frac{1}{2\sigma^{2}}{∥g_{t}-g_{t-1}∥}^{2}+V\left(g_{t},s_{t}\right)]-\log Z \end{array}$$ (6)

Here, u_*t_ ≡ *u*_*t*−1_ + L*(*g*_*t*_−*y*_*t*_) is the the predicted control and *ϵ* and *σ* govern the control noise and goal diffusion, respectively. In what follows, we will also find it useful to define *β* ≡ *σ*^−2^ and *U*(*g*) ≡ − *σ*^2^ *V* in order to write log *p*(*g*) = −*βE*(*g*|*s*) − log *Z*′ with

$$\begin{array}{c} E\left(g|s\right)=\sum_{t}[\frac{1}{2}{∥g_{t}-g_{t-1}∥}^{2}+U\left(g_{t},s_{t}\right)] \end{array}$$ (7)

which results from marginalizing the *u*_*t*_ out of Eq 6.

This formulation admits multiple interpretations: mostly simply, *E*(*g*|*s*) is the negative log probability of the goal time series: configurations that minimize this “energy” have higher probability. Along the same lines, the first term in Eq 7 is a penalty on large changes in *g* between time points, encouraging smoothness. In form, it is equivalent to a “kinetic energy” $K\equiv {\dot{g}}^{2}/2$. Likewise, the second term, *U*(*g*), is a generator of changes in goal state. At each time point, goals are drawn toward regions of small *U*, making it analogous to a “potential energy.” Together, the probabilistic model over goal trajectories in Eq 7 is equivalent to a path integral for a particle with position *g*_*t*_ and energy *K* + *U*. In the limit of small *V*/small *σ*/large *β*, one is in either the low-temperature thermodynamic limit or the high-mass classical limit, and *g* is a spatially-varying perturbation of a Gaussian process. Alternately, in the limit of large *σ*, goals are simply chosen independently at each time point. In any case, we have made the strong assumption that dependence of *g*_*t*_ on *g*_*t*−1_ occurs only through a momentum term, requiring that the “static” *V* term carries most of the weight of explanation.

Unfortunately, for general *V*(*g*|*s*), the distribution implied by Eq 7 is of the Boltzmann-Gibbs form and impossible to sample efficiently. If the goal of our inference is to model *V* itself, we will need a method for sampling from *p*(*g*) that still allows this partitioning.

#### *V* as a mixture of Gaussians

From the quadratic form of Eq 7, it is clear that one choice that leads to easy sampling is to assume that *e*^*V*^ is a Gaussian mixture model (GMM) at every time *t*:

$$\begin{array}{c} e^{V(g)}\propto \sum_{k=1}^{K}w_{k}[e^{-\frac{1}{2\sigma^{2}}{\left(g-\mu_{k}\right)}^{⊤}\cdot \Lambda_{k}\cdot \left(g-\mu_{k}\right)}\frac{|\Lambda_{k}|}{\sqrt{2\pi \sigma^{2}}}] \end{array}$$ (8)

with $\sum_{k=1}^{K}w_{k}=1$. Here, we write *μ*_*k*_ and Λ_*k*_ for the mean and precision matrices (inverse covariances) of the component Gaussians, and we have chosen not to absorb factors of *σ*^2^ into the precisions in order to have a common scale for ‖*g*_*t*_ − *g*_*t*−1_‖ and the eigenvalues of Λ^−1^. In practice we restrict ourselves to diagonal precision matrices, Λ_*k*_ = diag(**λ_*k*_**).

Given the assumption Eq 8, we can then write the evolution of *g*_*t*_ as a mixture of *K* terms, each of which takes the form

$$\begin{array}{c} p\left(g_{t}|g_{t-1},\mu_{t},\lambda_{t},k\right)\propto \exp(-\beta \sum_{j}[\frac{1}{2}{\left(g_{t}-g_{t-1}\right)}_{j}^{2}+\frac{\lambda_{kjt}}{2}{\left(g_{t}-\mu_{kt}\right)}_{j}^{2}]) \end{array}$$ (9)

where *j* indexes the dimensions of *g*. From this, it is clear that the conditional distribution of *g*_*t*_ is itself a mixture of Gaussians, which suggests the following method for sampling future goals:

$$\begin{array}{cc} k & ∼\text{Categorical}(w)\\ g_{t}|g_{t-1},\lambda_{t},\mu_{t},k & ∼N(\frac{g_{t-1}+\lambda_{kt}\mu_{kt}}{1+\lambda_{kt}},\frac{\sigma^{2}}{1+\lambda_{kt}}) \end{array}$$ (10)

where all multiplications and divisions by *μ*_*k*_ and λ_*k*_ are elementwise. That is, conditioned on previous goal position, sampling a new goal can be done by first sampling a particular component index *k*, then sampling from a Gaussian that interpolates between the kinetic and potential terms of the energy.

Clearly, assuming that *e*^*V*^ is a GMM at every time requires specifying (2*D* + 1)*KT* parameters per agent for *μ*_*tkj*_, λ_*tkj*_, and *w*_*tk*_, which can easily lead to overfitting. In our model, we contrain the form of *V* by requiring that these parameters are the outputs of a single neural network that takes the current state as its input: (*μ*_*kj*_, λ_*kj*_, *w*_*k*_)_*t*_ = NN(*s*_*t*_). In practice, the outputs of the network are unconstrained, with the tensor entries corresponding to λ and *w* subsequently passed through softplus and softmax functions (respectively) to ensure λ > 0 and ∑_*k*_ *w*_*k*_ = 1.

### Initial conditions

Let *t* = 1 be the time of the first observed data, *y*_1_. In order to calculate *y*_2_ using Eq 2, we need *u*_1_, which from Eq 3 requires *u*_0_, *e*_1_, *e*_0_, and *e*_−1_. (Recall that it is the joystick position *υ*_1_ that is observed; *u*_1_ is latent).

We bootstrap this process by assuming the following:

1. *y*_*t*≤0_ = *y*_init_, the vector of initial positions of both players.
2. For *t* < 0, *g*_*t*<0_ = *y*_0_, so there was no control error (*e*_*t*<0_ = 0) and thus no control (*u*_*t*<0_ = 0).
3. *g*_0_ ∼ GMM(*K*_0_). That is, the initial goal is drawn from a Gaussian mixture model with *K*_0_ components. In our case, these component Gaussians, like those defining Eq 8, have diagonal covariance.
4. From point 2 above, *e*_−2_ = *e*_−1_ = 0. Using *e*_0_ = *g*_0_−*y*_0_ then gives *u*_0_ via Eq 3.
5. Using Eq 10 and *g*_0_, we can then draw *g*_1_ and calculate *e*_1_ = *g*_1_ − *y*_1_. From Eq 3, it is then possible to calculate *u*_1_ and thus *y*_2_.

All future data points can then be calculated via the steps outlined above.

### Inference

Given the observed system trajectory *y*_*t*_, we would like to infer the underlying goal trajectory *g*_*t*_. In general, full Bayesian inference is intractable, but we employ a variational Bayes (VB) approach [19, 21] that approximates this procedure. In brief, VB attempts to minimize the Kullback-Leibler divergence (a measure of difference between distributions) between a known generative model for which inference is intractable, p(D,z), and an approximating family of posterior distributions, *q*(*z*). This is equivalent to maximizing an evidence lower bound (ELBO) given by

$$\begin{array}{c} L=E_{q(z)}\left[\log p\left(D,z\right)\right]-H\left[q\left(z\right)\right] \end{array}$$ (11)

with H[q(z)] the entropy of the approximating posterior. That is, inference is transformed into an optimization problem in the parameters of the approximate posterior *q*(*z*), amenable to solution by gradient ascent. In our model, we make use of so-called “black box” methods [22–25] in which the gradients of the ELBO are replaced with stochastic approximations derived by sampling from *q*(*z*), avoiding often difficult computations of the expectation in Eq 11. Thus our only requirement for the recognition model is that we be able to sample z_*_ ∼ *q*(*z*) and to compute log *q*(z_*_).

In our case, we begin with the generative model specified by Eq 6. For the approximate posterior *q*(*g*|*y*), we use the variational latent dynamical system (VLDS) model of [26, 27]. (We are not interested in the posterior over *u* and implicitly marginalize over it). The VLDS is a nonlinear generalization of the linear state space model, implying that inference in the model is a generalization of the Kalman filter. It uses neural networks to flexibly parameterize the mean and covariance of the underlying time series, which are assumed to change dynamically. As in [24, 25], samples from this posterior are then used to update *both* the parameters of the generative model (*L*, *ϵ*, *σ*, *μ*, λ, *w*) and the parameters of the approximate posterior *q* via gradient ascent.

#### Training

We implemented our model in TensorFlow (https://www.tensorflow.org) using the Edward probabilistic programming framework [28] (http://www.edwardlib.org). For the generative model of each agent, we set *K* = *K*_0_ = 20 and used a feed-forward neural network consisting of two 128-unit hidden layers with ReLU (Rectified Linear Unit) activation and a 128-unit dense output layer. For the posterior of *g*, we used a three-layer neural network with 64 units in each layer for the mean and two networks of the same structure for Cholesky factors of the diagonal and off-diagonal blocks of covariance matrices. We initialized all the weights in neural networks with Glorot uniform initializer [29] and all the biases with zeros. We fit the model using ADAM [30] with an initial learning rate of 10^−3^. To avoid overfitting, we held out 15% of the data (∼1300 trials) as a test set to check model fitting and to estimate the evidence lower bound. All model comparisons were performed on this test set.

To deal with the fact that the deterministic part of our control model, Eq 4, allows a solution *g* for any given *L*, and to encourage solutions near proportional control, we trained for an initial period of 30 epochs with the control parameters fixed to pure proportional control ((*K*_*p*_, *K*_*i*_, *K*_*d*_) = (1, 0, 0)). Following this, we allowed the control parameters to vary and trained for a subsequent 500 epochs, during which the percent change in a smoothed version of the ELBO dropped below 1% and parameter values stabilized.

For hyperparameter training we fixed *σ* ∼ 10^−3^ and regularized *ϵ* by including a penalty term −*ρϵ* with *ρ* = 10^5^ (equivalent to an exponential prior) to the ELBO. As described above, when joystick inputs were near the end of their physical range, control and goal estimates could exhibit large variation due to inversion of the tanh function. We regularized this by including a hinge loss that linearly penalized modes of the GMM when they exceeded the game area by 50% in any direction. (The performance of the model without this penalty can be found in S1 Fig). Finally, we regularized Gaussian components of the GMM by penalizing those with very large standard deviation. That is, we added a penalty −*γ*/λ_*kt*_ with *γ* = .1 at each time point, which is equivalent to placing an exponential prior on the variance.

### Change point analysis

We used the number of change points in the puck trajectory as a rough measure of the each player’s strategic complexity. Specifically, a time point *t* is a change point for a player if sign(*υ*_*t*_) ≠ sign(*υ*_*t*+1_), *υ*_*t*_, *υ*_*t*+1_ ≠ 0, and |*υ*_*t*_ − *υ*_*t*+1_| ≥ 10^−6^. That is, joystick input changes sign, is nonzero both before and after the change point, and the difference exceeds a minimal value. We studied the correlation between number of change points and game results at both the trial and session level. To avoid over-identification of change points due to frequent variation in control signals, all trials were smoothed by the same Gaussian filter described in Data section before this analysis. We also excluded the first and last five time points to minimize edge effects.

### Statistical testing

For testing autocorrelation functions, we applied the Ljung-Box test to the autocorrelation function calculated on each session. For initial velocities, we discarded periods of non-movement at the start of the trial, selecting instead the velocity at the first time point where the norm of the velocity exceeded 0.001. For analyzing whether outcomes of consecutive trials were correlated, we used Fisher’s Exact Test on game results (summarized by a 2 × 2 contingency table of win/loss of *t*^th^ and (*t* + 1)^th^ trials) by session. We corrected for multiple comparisons via Bonferroni correction to ensure a family-wise false positive rate of 1%.

## Results

### Player tendencies

**Fig 2:**
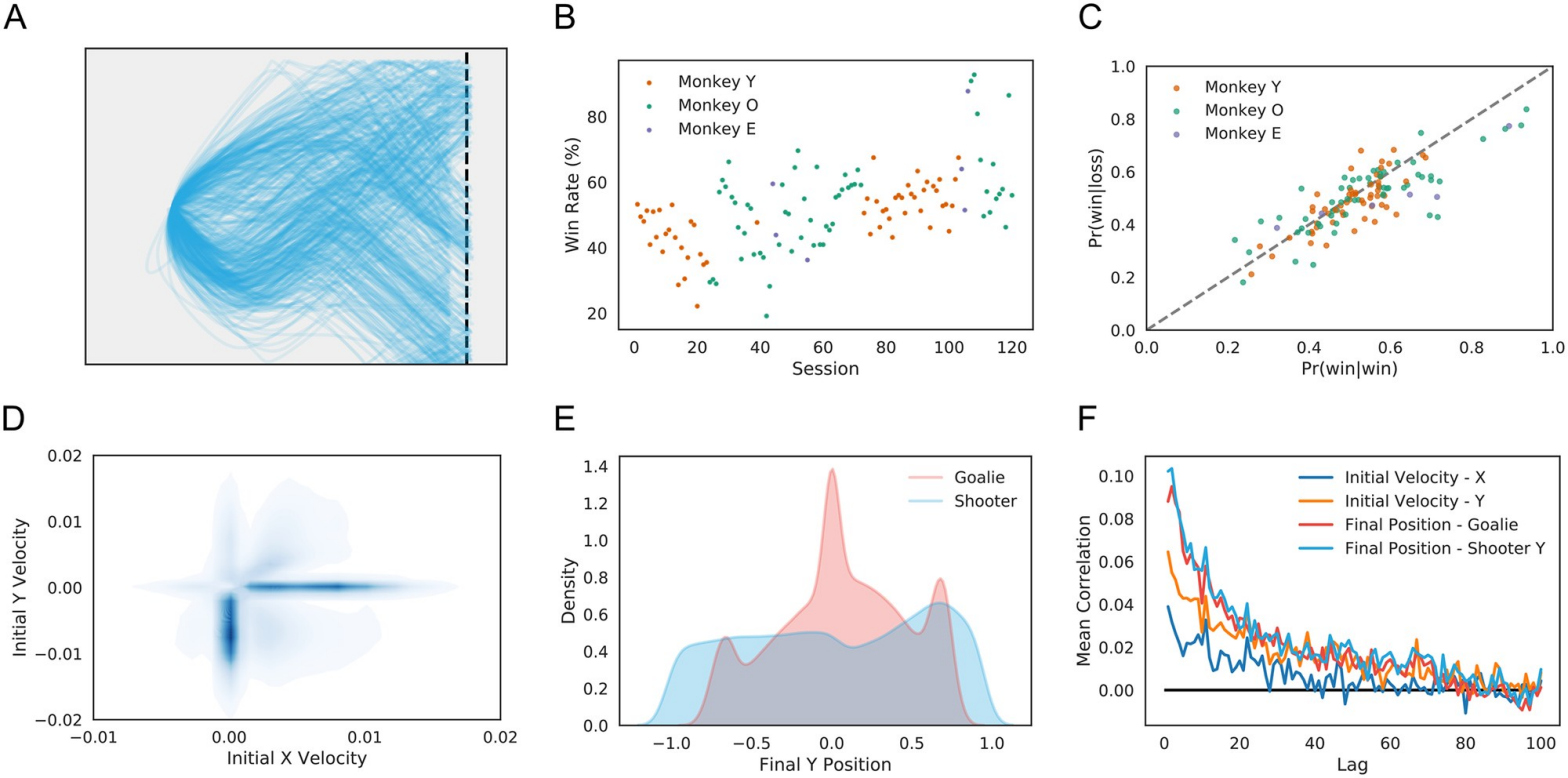
Variability in player behavior. A: Example trajectories from a single shooter, illustrating the diversity of player movements. B: Percentage of trials won by shooter for each behavioral session. Colors indicate shooter identity. Task parameters (puck speed, goalie size) were altered on a per-session basis to balance game play between players. C: Correlations in game outcome. Scatter plot of the probability of a shooter win as a function of shooter win or loss on the previous trial. Each dot represents one behavioral session. Colors indicate shooter identity. Game outcomes did not differ markedly following wins or losses. D: Distribution of initial velocities over all shooters. Shooters tended to start by moving immediately rightward, toward the goal line, or directly downward. E: Distribution of final vertical positions. Final goalie positions were peaked around 0 and either end of the screen, while shooter final positions were more evenly distributed over the game space. F: Autocorrelation in initial movement and final position across trials. Shooters’ initial movement direction and both players’ final vertical positions exhibited weak correlation across trials, indicative of short-term patterns in strategy. Curves are averages across behavioral sessions. Colors indicate variable.

While players’ behavior was highly variable across trials and sessions (Fig 2A), blockwise adjustments to players’ maximum velocity kept win rates similar across players (Fig 2B). Likewise, the likelihood of a win was independent of whether the previous trial was a win or a loss (*N* = 4/120 sessions significant for dependence; Fisher’s Exact Test for independence; *α* = 0.01), suggesting little in the way of a “win-stay, lose-shift” or “hot hand” effect (Fig 2C). Moreover, while players’ initial velocities tended to cluster, their final positions were more evently distributed (Fig 2D and 2E). That is, players appeared to mix their play effectively. We found that initial velocities and final positions were only weakly autocorrelated (*v*_0*x*,shooter_ = 4/120, *v*_0*y*,shooter_ = 17/120, *y*_*f*,goalie_ = 21/120, *y*_*f*,shooter_ = 31/120 sessions significant; Ljung-Box test for autocorrelation at lag 1; *α* = 0.01 controlled for family-wise error rate) (Fig 2F), indicating that there was little strategic carryover across trials. In summary, while significant complexity and variation in play is apparent, this variation was not well captured by typical metrics derived from games with discrete action spaces.

### A generative model of player behavior

**Fig 3:**
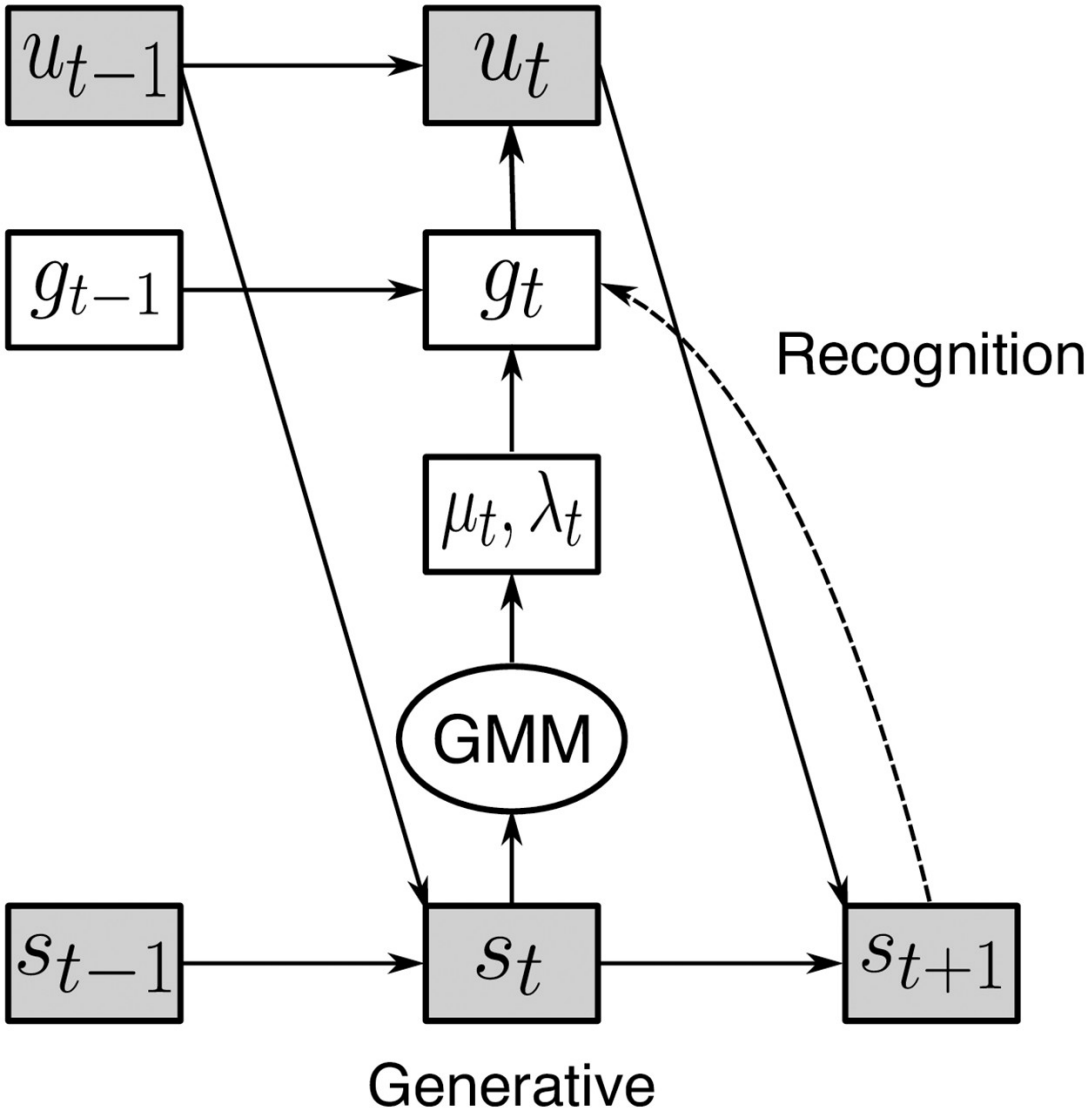
Model architecture. Observable trajectories *y*_*t*_ are generated sequentially from control signals *u*_*t*_, which in turn derive from goals *g*_*t*_. In the generative model, evolution of goals is determined by a Gaussian mixture model whose parameters are given by the output of a neural network using state variables *s*_*t*_ as input. The recognition model takes in the lagged history of *y*_*t*_ and returns posterior samples of the goals.

Because play in the penalty shot task is fundamentally dynamic and interactive, it is resistant to conventional models based on either discrete action spaces or simple heuristics. Instead, we opted to model player behavior as arising from two pieces: first, a dynamic goal model that encoded each player’s desired onscreen location as a function of both players’ instantaneous positions and velocities; and second, a control model that used these goals as its set points and produced joystick movement (Fig 3). Goals evolved dynamically with the game. This was captured in the model by a “value function” that at each moment drew each player’s desired onscreen location toward areas of high value and away from those of low energy. (This is not the same as the value function in reinforcement learning, which sums all future rewards. Our *V* is more closely related to policy than expected value). For example, with the goalie at the upper edge of the screen, the energy function for the puck is expected to be low at the lower edge of the screen. However, it is important to note that energy functions for each player were separate. While both made use of explicit information from both players (positions and velocities), each player’s goals were private (i.e., independent of one another given current game state).

**Fig 4:**
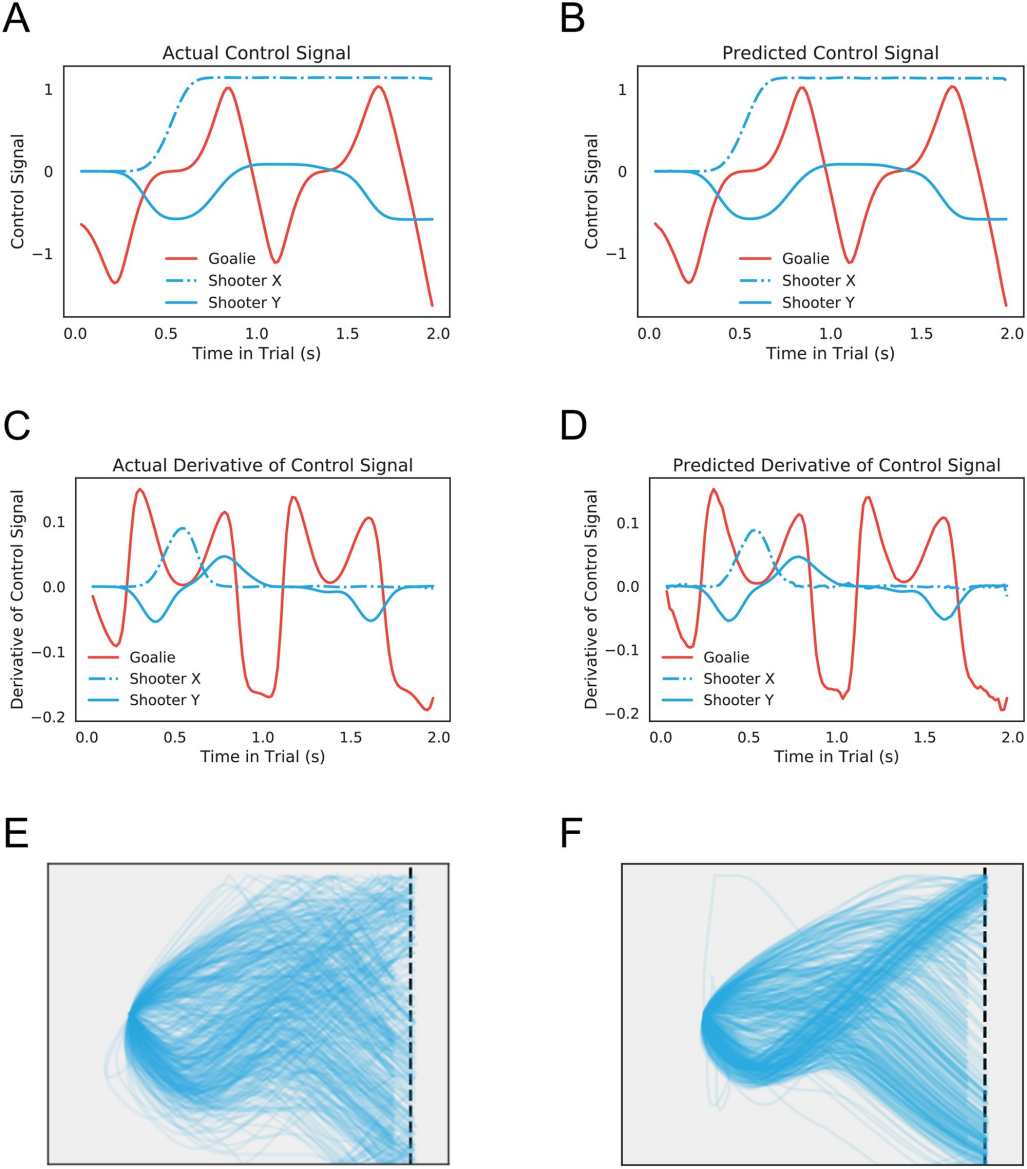
Model fits and generated data. A: Actual control signals for both coordinates of puck (blue) and bar (red) for a single trial in the holdout set. B: Model predictions for control signals at the next time step given real data up to the current time. The fitted model’s predictions control closely track the real data. C, D: Plots of actual control derivatives and model-predicted derivatives for the same trial. On this scale, deviations become more apparent, but the model still closely fits the data. In both cases, model fits reflect the accuracy of the posterior map, not necessarily the generative model. E: Real puck trajectories (n = 500) drawn from a model fit to the last ten sessions of the experiment (only trials that last less than $\frac{25}{6}\text{s}$ are displayed, though longer trials were included in training). F: Puck trajectories (n = 500) generated from the model (only trials that last less than $\frac{25}{6}\text{s}$ are displayed). The model has accurately captured the qualitative features of real play.

We trained this model using all behavioral sessions of the task as input. To account for variation in play across the experiment, we included a term that increased linearly as a function of session, as well as additional variables encoding experimental conditions (see Methods). Fig 4 shows results from a restricted model using only the last ten sessions of data from a single shooter. Not only is the model able to produce accurate one-step-ahead predictions of the control signal (Fig 4A) and its derivative (Fig 4B), it is able to generate synthetic data that capture the rich behavioral repertoire exhibited by real players (Fig 4C and 4D).

**Fig 5:**
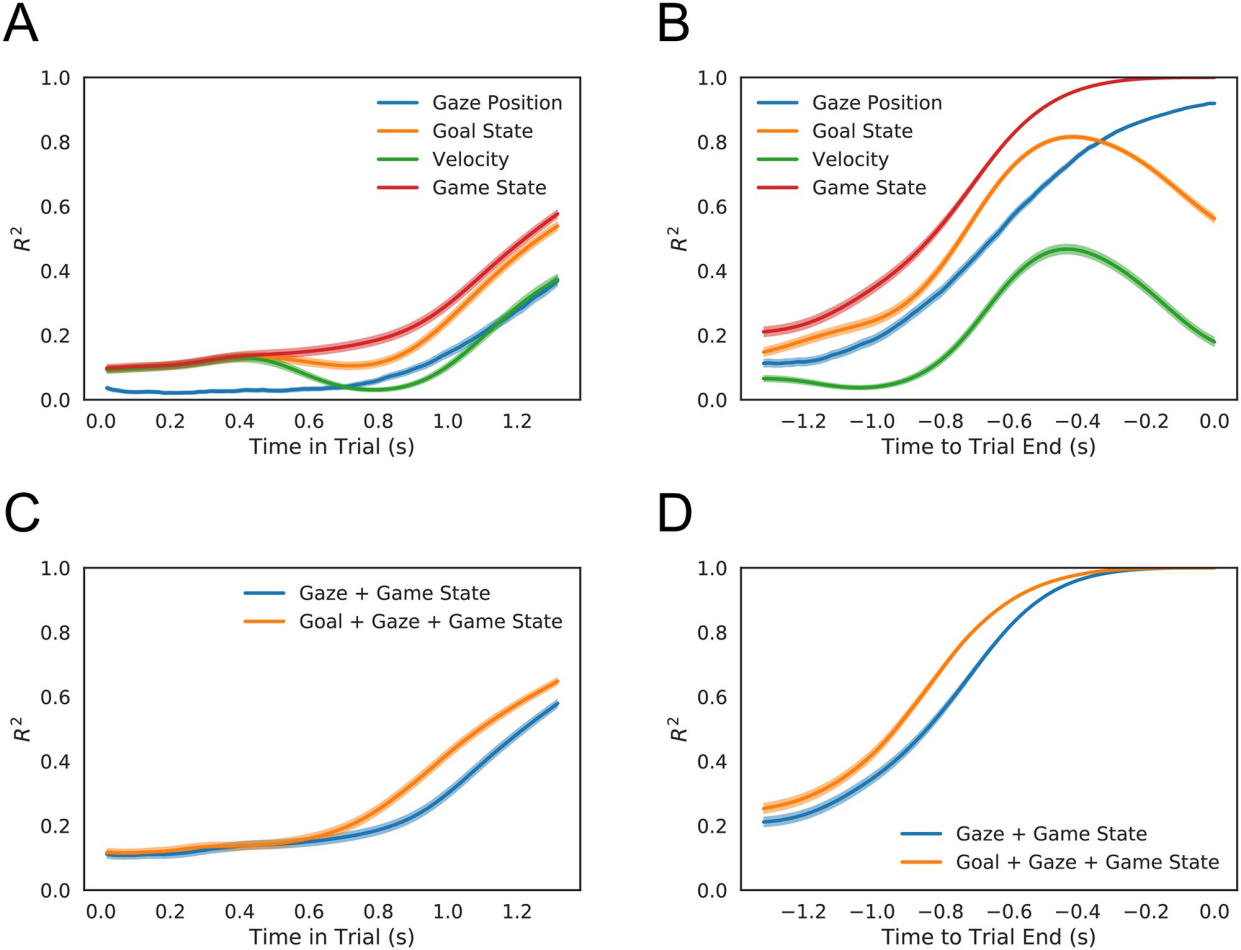
Inferred goals predict game endpoints. A, B: Predictive power (as measured by *R*^2^) for a model predicting final puck position as a function of either shooter gaze position, instantaneous velocity, game state, or inferred goal. C, D: Predictive power for a model predicting final puck position as a function of all variables with or without inferred goals. A, C: Aligned to trial start. B, D: Aligned to trial end. Game states consistently outperform the other variables. Inferred goals are just as predictive as the velocity of movement for the first 0.4s, but become more predictive thereafter. They remain more predictive than either measure until 0.3s before trial end, when gaze becomes a better predictor. In general, including inferred goal states in the model enhances the predictive power.

To assess the utility of our inferred goals as a construct, we next asked whether these goals contained predictive information about gross strategic behavior. To do so, we used a simple linear regression of final puck vertical position against goal position at each moment of each trial and calculated the coefficient of determination (*R*^2^) as a function of time in trial. Fig 5 shows this function time-locked to both the beginning and end of trial. For comparison, we repeated the same analysis using two other predictors, the puck’s instantaneous velocity and the shooter’s eye position. We found that the shooter’s goal became more predictive than the puck’s velocity within the first 0.5s of the trial (Fig 5A) and remained the most predictive of the three variables until the last 0.3s of the trial, when it was overtaken by gaze (Fig 5B). As might be expected, gaze becomes the most predictive single variable late in trial (players attend to the location where the outcome will be determined), while variables like velocity and goal, which only influence position after a lag, become slightly less accurate. Moreover, while state, comprising both the players’ positions and velocities, is more predictive than any single variable, including gaze, adding goal information to state information does improve predictive performance (Fig 5C and 5D). This rough analysis is thus consistent with the idea that our inferred goals provide information not contained in state and can be treated as a latent measure of momentary player intentions.

### Model comparison

**Fig 6:**
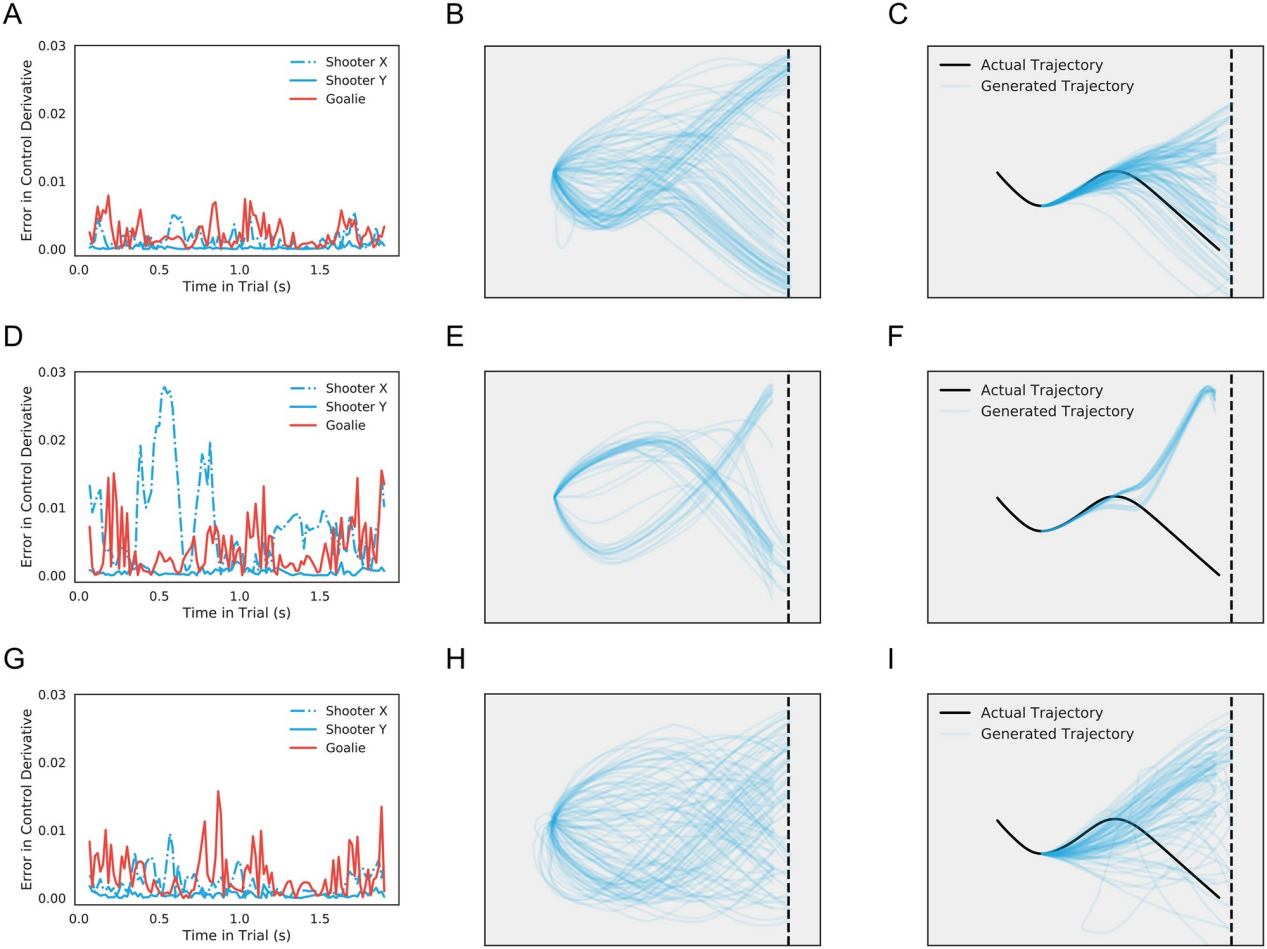
Model comparison. A: Difference between actual and predicted control derivatives for a trial (the one in Fig 4) in all three observed dimensions in the proposed model. B: Puck trajectories (n = 100) generated from the proposed model (only trials that last less than $\frac{25}{6}\text{s}$ are displayed). C: Completed trajectories (n = 100) by the proposed model from $\frac{7}{15}\text{s}$ of a trial in the holdout set (only trials that last less than $\frac{25}{6}\text{s}$ are displayed). D, E, F: the same figures based on the model with single Gaussian distribution. G, H, I: the same figures based on the linear model.

Our assumption of a Gaussian mixture parameterized by neural networks in Eq 8 is a highly flexible one, raising the question of whether our model simply memorizes trajectories or whether a simpler set of assumptions might do. Fig 6 shows the results of comparing our model to two simpler variants on tests of trial generation and trial completion. The first comparison model assumes the same neural network structure but with a single Gaussian output distribution at each time *t*:

$$\begin{array}{c} e^{V(g)}\propto e^{-\frac{1}{2\sigma^{2}}{\left(g-\mu \right)}^{⊤}\cdot \Lambda \cdot \left(g-\mu \right)}\frac{\left|\Lambda \right|}{\sqrt{2\pi \sigma^{2}}}; \end{array}$$ (12)

The other comparison model uses a linear function in place of a neural network to model the dependency of goals on state:

$$\begin{array}{c} \begin{array}{cc} \mu_{t} & =W_{1}s_{t}+b_{1}\\ \lambda_{t} & =\text{softplus}(W_{2}s_{t}+b_{2})\\ w_{t} & =\text{softmax}(W_{3}s_{t}+b_{3}) \end{array} \end{array}$$ (13)

where {(*W*_1_, *b*_1_), (*W*_2_, *b*_2_), (*W*_3_, *b*_3_)} are weights and biases, softplus(*x*) = log(1 + *e*^*x*^), and $\text{softmax}{\left(x\right)}_{i}=e^{x_{i}}/\sum_{j}e^{x_{j}}$.

We trained all three models on the same dataset with the same hyperparameters and stopping criteria specified above and then compared their performances on fitting control signals and derivatives, generating new data, and completing trials. Our model outperformed the two candidates in all three aspects: the approximate posterior for our model yields the overall smallest prediction error (Fig 6A, 6D and 6G); the generated trajectories resemble the real ones the most closely (Fig 6B, 6E and 6H); and trial completion for the neural network GMM demonstrates the most diversity (Fig 6C, 6F and 6I). That is, the simpler models proved either insufficiently flexible to capture regularities in the data, resulted in quasi-deterministic behavioral policies, or both.

### Simulation experiments via generative modeling

By adopting a generative modeling approach along with a flexible class of parameterized functions (neural networks), we have shown that our model can capture the variability present in real data (Fig 4F). An additional benefit of such an approach is that our model can then be used as a simulator for purposes of running controlled experiments. These “counterfactual” simulations allow us to systematically vary task parameters and states to explore the responses of the trained model in configurations only sparsely covered by actual data.

**Fig 7:**
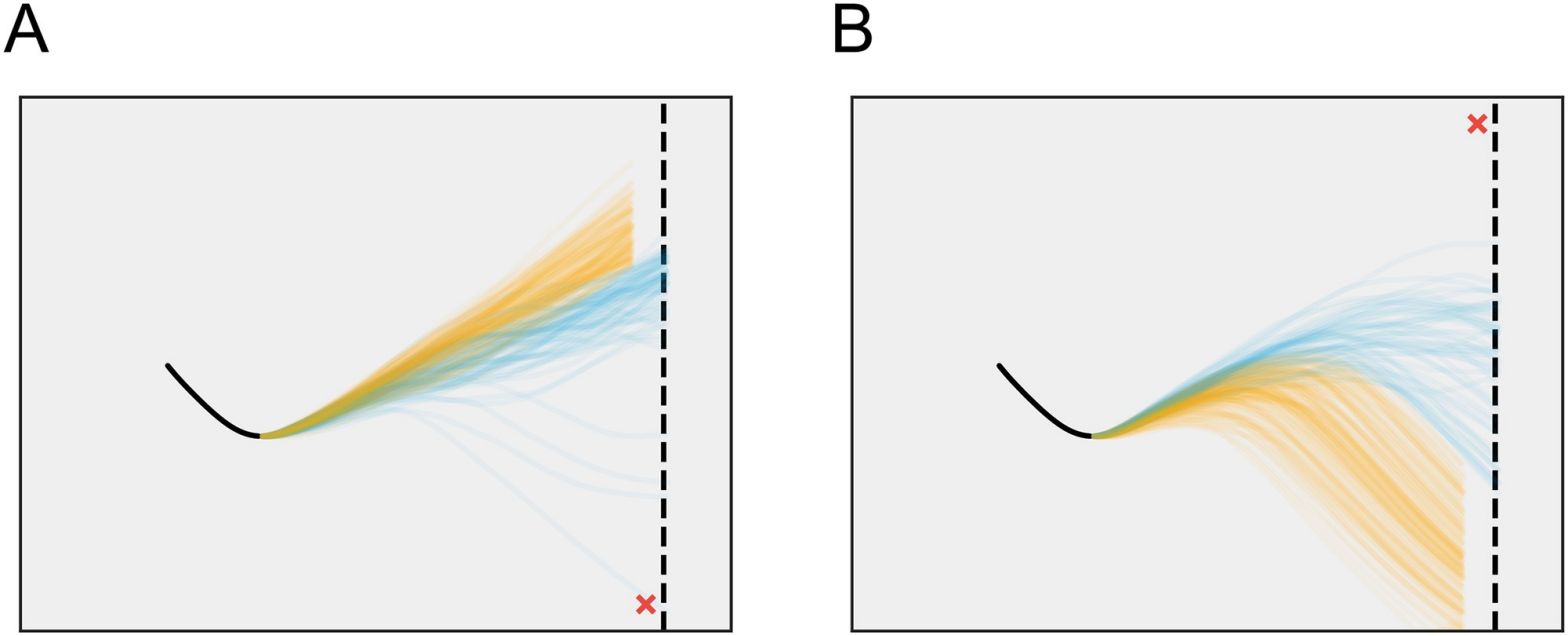
Effects of goal states. A: Completed puck trajectories (n = 200) for the trial in Fig 6C with the goal state at $\frac{7}{15}\text{s}$ for goalie is set at the bottom of the screen (only trials that last less than $\frac{25}{6}\text{s}$ are displayed). B: Completed puck trajectories (n = 200) for the same trial with the goal state at $\frac{7}{15}\text{s}$ for goalie is set at the top of the screen (only trials that last less than $\frac{25}{6}\text{s}$ are displayed).

Here, to illustrate the potential of this approach, we performed a simple experiment to explore the effects of goal states on trial outcomes. If, as we claim, goals capture players’ latent targets as a function of changing game state, then altering goals should change subsequent game play. To check this, we fixed a time point midway through a specific trial and performed a series of trial completion experiments (Fig 7). In one set of experiments, the goalie’s goal is fixed to the lower corner of the screen (Fig 7A, red X), while in the other, it is fixed in the upper corner of the screen (Fig 7B, red X). In both cases, the shooter’s goals are allowed to evolve normally, and the goalie’s goals are allowed to move normally after the first time point of the trial completion.

As Fig 7 illustrates, subsequent play is a product not only of initial goals, but of players’ co-evolving control strategies. As one expects, when the goalie’s initial goal is downward, the shooter compensates by moving the puck upward; when the goalie’s initial goal is upward, the shooter steers the puck down. However, what is more interesting to note is the subsequent trial outcomes: in Fig 7A, more extreme puck trajectories result in losses (orange), since these more clearly signal the shooter’s intention and prompt the goalie to reverse direction more quickly. Likewise, in Fig 7B, earlier downturns in puck trajectory also result in losses. In both cases, the initial goal is rapidly reversed from its location at the start of the simulation, while less extreme or more ambiguous trajectories are likelier to result in wins (blue). Thus, the results of our simple experiment suggest both that the model has done more than simply memorizing trajectories and that it can be used to answer “what if” questions via simulation.

### Quantifying strategic complexity

Since, as we have argued, players’ inferred goals constitute a moment-by-moment representation of strategic intention, and since the evolution of these goals is governed by each player’s value function *V*(*g*, *s*) (Eq 7), these components of our model are ultimately responsible for capturing the richness of players’ interactions. For instance, at a fixed time *t* in the trial, the structure of *V*(*g*_*t*_, *s*_*t*_) determines whether a player’s trajectories fall into groups that will split or merge, how variable the trajectories are within each group, and thus how complex the shooter’s corresponding strategy is.

**Fig 8:**
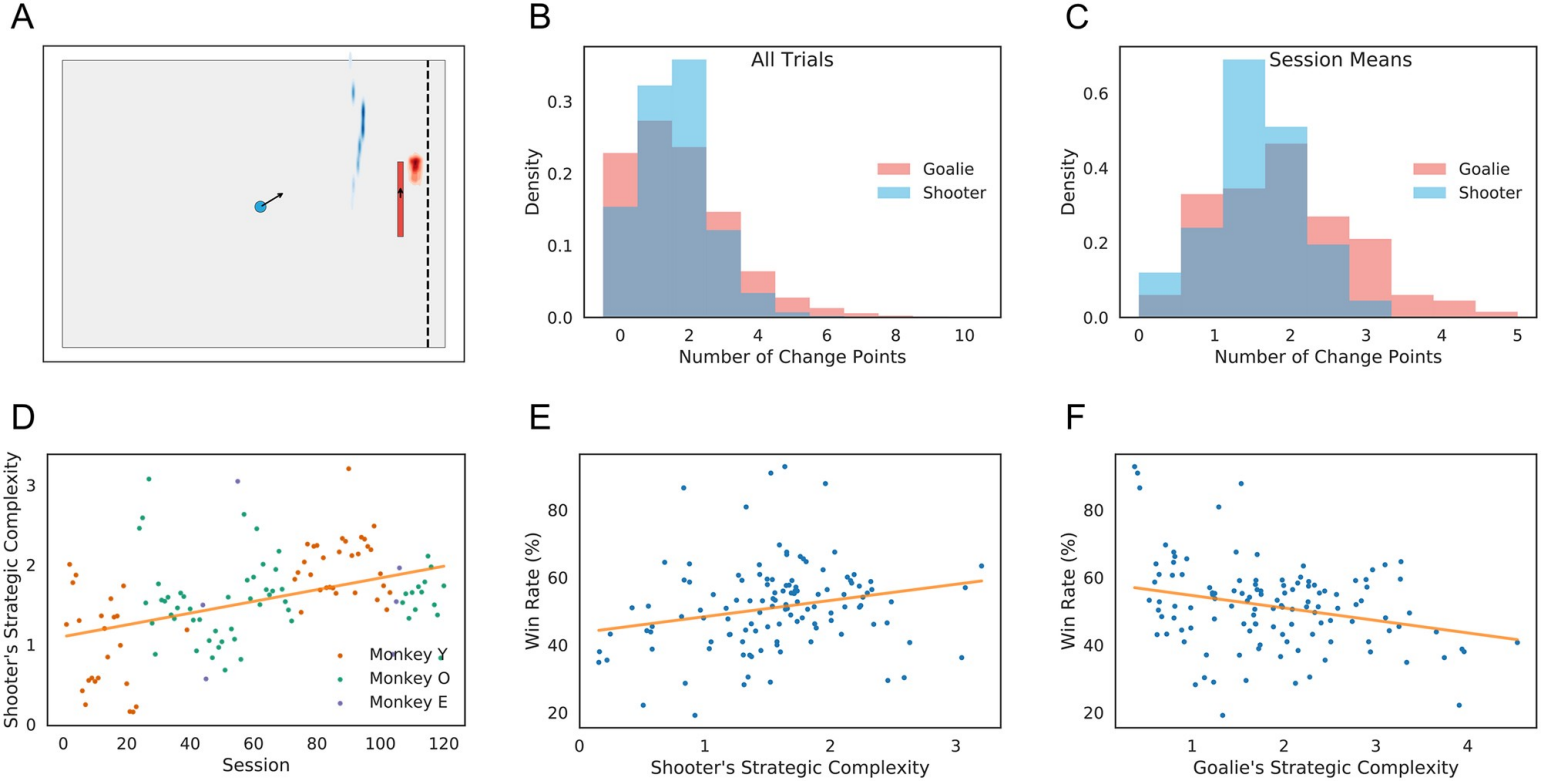
Number of change points as a measure of strategic complexity. A: At a given game state, each player’s energy function (blue, shooter; red, goalie) captures the evolution of his goal in the next time step. Here, the goalie’s energy function is unimodal, while the shooter’s energy function consists of a tightly clustered handful of potential targets. Gray area indicates the visible screen (game area) during the task. White indicates offscreen regions. We use the number of change points in each player’s control signal as a proxy for this complexity. B, C: Histograms of change points for all trials (B) and session means (C) for both players. D: Evolution of strategic complexity across sessions. Over the course of the experiment, the average number of shooters’ change points per trial increased. E, F: Correlation of average number of change points of shooter (E) and goalie (F) with shooter’s win rates. Each dot indicates a single session. Both shooters and goalies benefited from more change points.

Fig 8A depicts this strategic complexity at a moment midway through the trial. With the puck moving upward, the shooter’s value function (blue) is highest at a cluster of points spread vertically to the left of the goal line, indicating that the goals are pulled in that direction. Likewise, the value function for the goalie (red) is concentrated above the current vertical position of the puck, suggesting a tendency to follow its movement. As the form of these value distributions changes, so does the variability of the resulting trajectories.

To quantify this strategic complexity, we considered the number of direction changes (change points) in each player’s trajectory on each trial. With this definition of complexity, we see that variation in strategic complexity exists at both the trial (Fig 8B) and session level (Fig 8C). A one-way ANOVA shows significant differences across sessions for both shooters(*F*_119,59764_ = 128.950, *p* < 1.0 × 10^−6^) and goalies (*F*_119,59764_ = 216.990, *p* < 1.0 × 10^−6^). Moreover, the average number of shooter change points shows a weakly increasing trend across sessions (Fig 8D; *β* = 0.00735/session, Wald test *p* = 4.123 × 10^−8^), indicating that players adopted more complex strategies over time. As one would expect, an increase in number of change points, corresponding to an increase in the variability of trajectories, translates to an increase in win rate for both shooters (Fig 8E; *β* = 4.793%/change point, Wald test *p* = 3.381 × 10^−3^) and goalies (Fig 8F; *β* = 3.695%/change point, Wald test *p* = 8.896 × 10^−4^). Thus, players’ strategic complexity, as measured by the number of change points, can serve as a useful metric for evaluating player behavior.

## Discussion

In natural contexts, particularly social ones, decisions are made in real time and in response to continuously changing circumstances. Whereas most social tasks in neuroscience have focused on two-player games with sequential moves and small action spaces [3, 6, 11, 12, 31–34], here we have demonstrated that tasks in which opponents make decisions online, in real-time, are likewise amenable to principled computational analysis. The virtues of such tasks are many: greater ethological validity, greater involvement of sensory and motor systems typically involved in the decision process [35–37], and dynamics better aligned with the timescales of most neural circuits.

Nonetheless, nothing about our model presumes that the task is social (nor tests whether it is). We do not explicitly model players’ beliefs about one another, bounded rationality, or theory of mind [38, 39]. Some of these constructs may be implicit in our estimated value function, but teasing them apart is a topic for future work. As it stands, our model is empirical and agnostic as to the degree to which players model one another. This bears some similarity to work in differential game theory, which has established conditions under which moving target pursuit tasks like ours possess optimal solutions, as well as the expected values for each player under those strategies. [40, 41] As in that literature, we have adopted control theory as a guiding framework, with our control policies determined by *V*(*g*|*s*). In addition, we have made the simplifying assumption common to differential game analysis that players are coupled only through observable states *s*. Here, however, we limit ourselves to modeling players’ adopted strategies, without regard to whether or not these are optimal.

Our model is, in effect, learning a complex control policy in a six-dimensional state space. This policy differs among the individuals in the study and, as the result of a statistical fitting process, is unlikely to be successfully extrapolated to regions of state space in which no training data exist. It would be impressive if one were, for example, to omit all trials with an initial upward puck movement from the training data and later predict them from a model trained on the rest, but given the lack of symmetry along the vertical axis in the actual trials, we suspect this is simply not possible. Still, we have demonstrated that despite the richer data resulting from our task, our scalable Bayesian inference approach is sufficient to address the resulting analysis challenge. We believe our model provides a useful distillation of the data in terms of more intuitive variables, and that these variables contain some predictive and internal validity. Likewise, our choice of a probabilistic generative model for the data (as opposed to a discriminative model like a classifier) offers two key advantages: First, as a test of our model’s goodness of fit, we are able to generate synthetic data that capture much of the complexity observed in real data. So-called posterior predictive checks offer a higher level of confidence that models are detailed enough to account for variations in the data and not simply the best among poor alternatives [42]. Second, because our model uses only data from onscreen and the recent past in generating synthetic trials, it is capable of serving as an artificial opponent in future experiments. Such a model could be trained to reproduce the play of a particular opponent, allowing for an experiment in which the distinction is not between a conspecific and an unrelated computer algorithm, but between a conspecific and an algorithm with an indistinguishable style of play. Such contrasts are crucial for teasing apart differences specific to the social context [9, 11–14].

Moreover, our model succeeds in separating out details of the motor execution of the task (physics model, control model) from the dynamics of latent goals that forms the core construct of interest. For neural data with high temporal resolution, instantaneous measures of such quantities as intention, expected value, and strategic complexity—likely correlated with decision difficulty or cost of control [43]—are valuable as potential correlates of local circuit activity. As we have shown, variables like these are able to differentiate between trials, sessions, and players where more coarse-grained metrics cannot.

Finally, while the current model is broadly applicable to any time series data that can be viewed as arising from control applied to a dynamic set point, several worthwhile extensions are possible: First, we have not adequately treated the problem of censoring of the control signal. That is, the joystick position is a truncated version of each player’s desired control signal. A more natural model would model and correct for the resulting missing data. Second, we have modeled the value function that drives changes in goal as a mixture of Gaussians at each time step. While this proved adequate for our purposes, it would be natural to consider an extension in which this function is replaced by a Hidden Markov Model (or hierarchical HMM) with Gaussian observations, so that trials are generated by goals that follow a single mode (perhaps corresponding to a substrategy) for short periods of time.

### Conclusion

The study of complex strategic decisions, especially social decisions, demands experimental paradigms that replicate this complexity. Here, we have introduced a two-player computerized task, Penalty Shot, that requires real-time behavioral adjustment and facilitates strategic variation. Not only do pairs of rhesus macaques readily learn the game, they exhibit individual playing styles and complex patterns of interactive play. By using a generative modeling approach that leverages approximate Bayesian inference and modern gradient-based training methods, we have shown that it is possible to capture this variability and produce moment-by-moment estimates of latent intentions and strategic complexity suitable for correlation with neural data. These results open new possibilities for the analysis of dynamic behavior and its associated neural data and have implications for the study of social behavior.

## Supporting information

S1 Fig Without regularization, goals far exceed game area. A: Difference between actual and predicted control derivatives for the same trial analyzed in the Model comparison section. B: Predicted goal states in all three observed dimensions. C: At 0.5s, both players’ energy functions (blue, shooter; red, goalie) based on this model are far off the game arena (gray).
(TIF)

S2 FigModel comparison: Trial completion.For 10 random trials in the validation data set, we completed trajectories (n = 100) using the proposed model, the single Gaussian model, and the linear model. The actual trajectories are in black, and the completed ones are in blue. Only trials that last less than $\frac{25}{6}\text{s}$ are displayed except the single Gaussian model.(TIF)

S3 Fig Generated trials matched with actual trials. For 30 random trajectories generated by our model, we located and plotted the closest 20 trials (in mean-squared state space error) in the training data set. The generated trajectories are in black and the matched ones are in blue (the most similar one in orange).
(TIF)

S1 Video Single trial animation. Animation of a real single trial played by two subjects, the same trial with goals overlaid, and a third presentation of the same trial with energy function overlaid as a heat map.
(MP4)

S2 Video Single trial animation. (MP4)

S3 Video Single trial animation. (MP4)

S4 Video Single trial animation. (MP4)
