# Supplementary figures and images for: Latent goal models for dynamic strategic interaction
Source: PLoS Comput Biol. 2019 Mar 11;15(3):e1006895. doi: 10.1371/journal.pcbi.1006895 (PMC6472832; doi:10.1371/journal.pcbi.1006895)

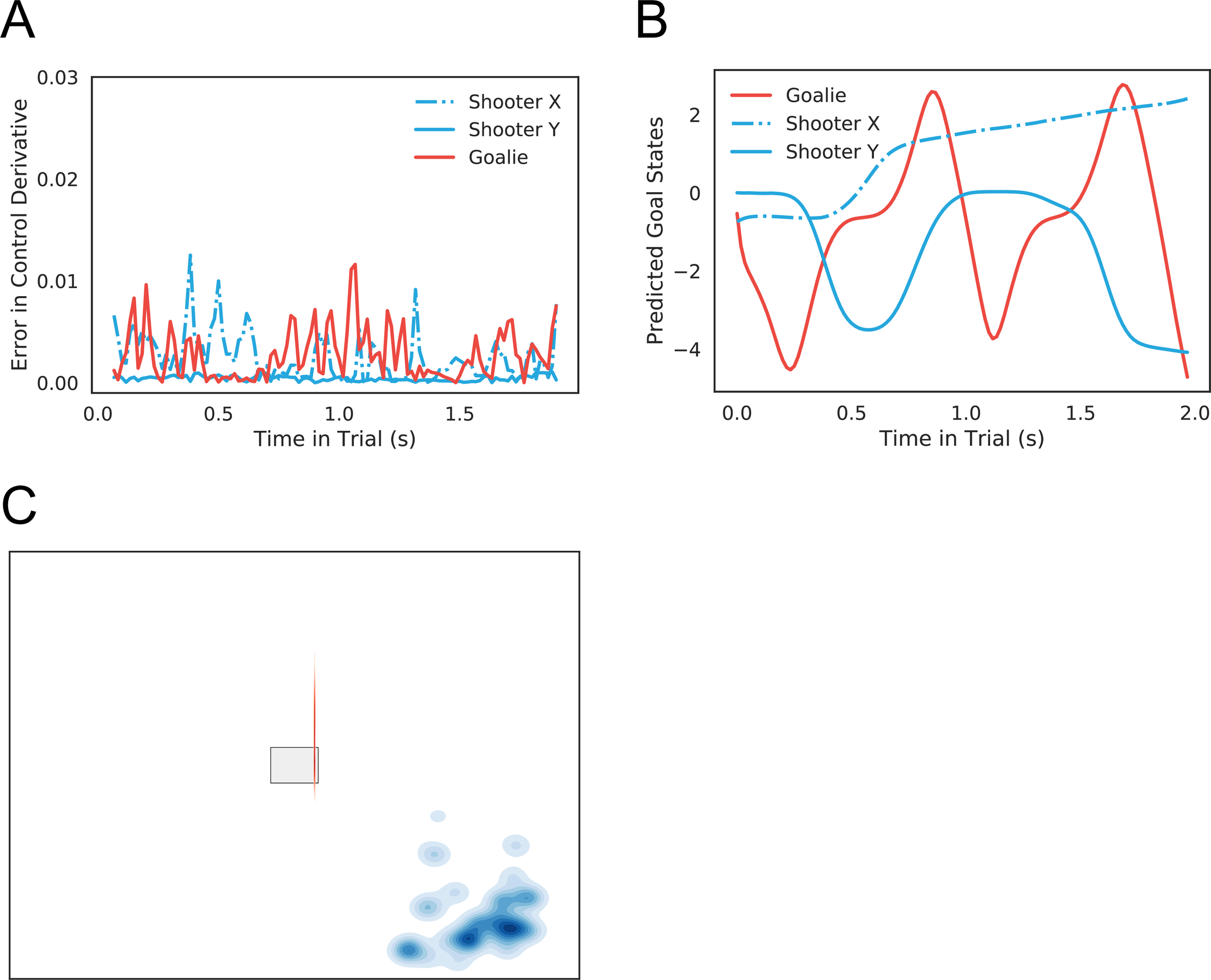

Supplement: S1 Fig — A: Difference between actual and predicted control derivatives for the same trial analyzed in the Model comparison section. B: Predicted goal states in all three observed dimensions. C: At 0.5s, both players’ energy functions (blue, shooter; red, goalie) based on this model are far off the game arena (gray). (TIF) [file pcbi.1006895.s001.tif]

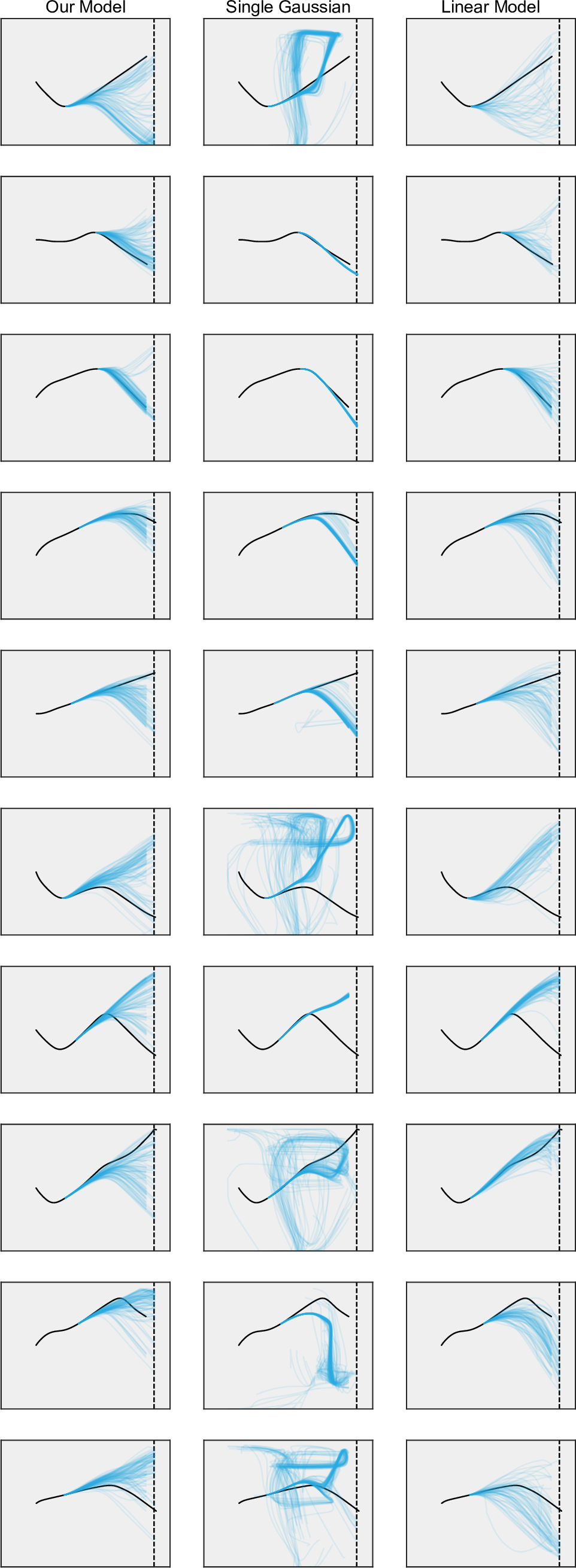

Supplement: S2 Fig — For 10 random trials in the validation data set, we completed trajectories (n = 100) using the proposed model, the single Gaussian model, and the linear model. The actual trajectories are in black, and the completed ones are in blue. Only trials that last less than 256s are displayed except the single Gaussian model. (TIF) [file pcbi.1006895.s002.tif]

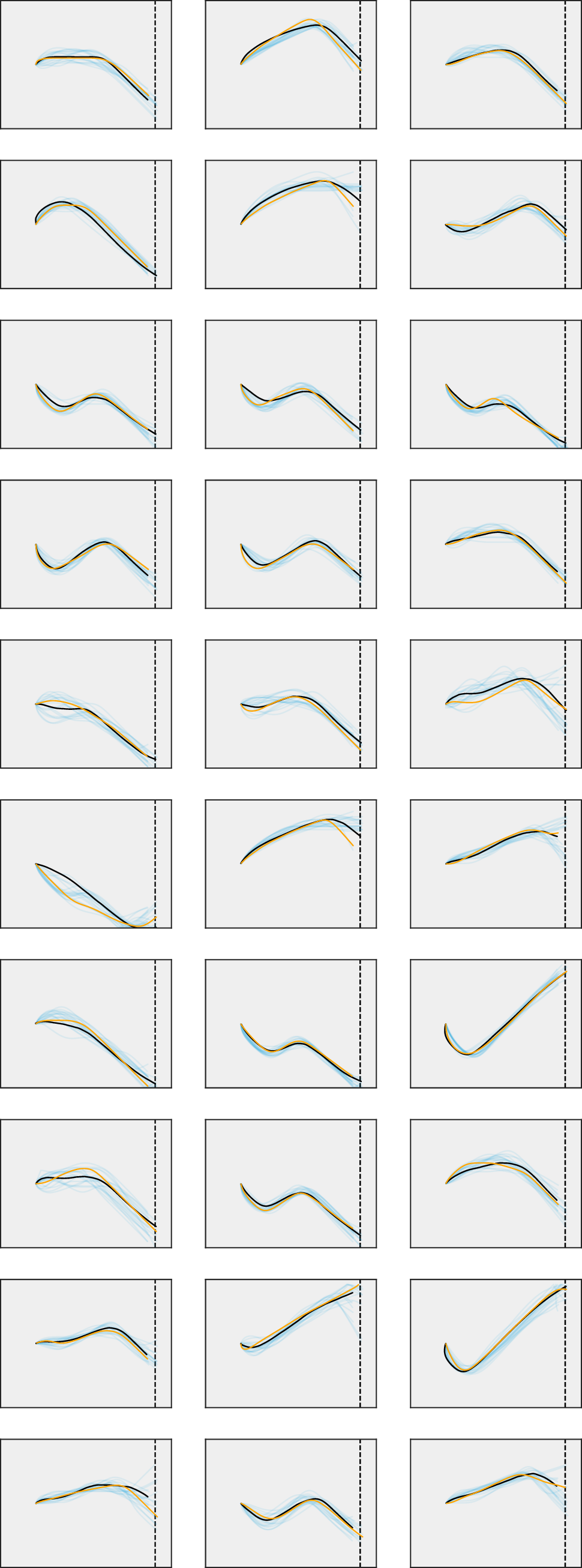

Supplement: S3 Fig — For 30 random trajectories generated by our model, we located and plotted the closest 20 trials (in mean-squared state space error) in the training data set. The generated trajectories are in black and the matched ones are in blue (the most similar one in orange). (TIF) [file pcbi.1006895.s003.tif]
